# APOE Polymorphism Affects Brain Default Mode Network in Healthy Young Adults: A STROBE Article

**DOI:** 10.1097/MD.0000000000001734

**Published:** 2015-12-31

**Authors:** Yun Yan Su, Xue Liang, U. Joseph Schoepf, Akos Varga-Szemes, Henry C. West, Rongfeng Qi, Xiang Kong, Hui Juan Chen, Guang Ming Lu, Long Jiang Zhang

**Affiliations:** From the Department of Medical Imaging, Jinling Hospital, Medical School of Nanjing University, Nangjing, Jiangsu Province, China (YYS, XL, RQ, XK, HJC, GML, LJZ); and Division of Cardiovascular Imaging, Medical University of South Carolina, Ashley River Tower, Charleston, South Carolina (UJS, AV-S, HCW).

## Abstract

To investigate the effect of apolipoprotein E (*APOE*) gene polymorphism on the resting-state brain function, structure, and blood flow in healthy adults younger than 35 years, using multimodality magnetic resonance (MR) imaging.

Seventy-six healthy adults (34 men, 23.7 ± 2.8 y; 31 *APOE ε4/ε3* carriers, 31 *ε3/ε3* carriers, and 14 *ε2/ε3* carriers) were included. For resting-state functional MRI data, default mode network (DMN) and amplitude of low-frequency fluctuation maps were extracted and analyzed. Voxel-based morphometry, diffusion tensor imaging from structural imaging, and cerebral blood flow based on arterial spin labeling MR imaging were also analyzed. Correlation analysis was performed between the above mentioned brain parameters and neuropsychological tests.

There were no differences in neuropsychological performances, amplitude of low-frequency fluctuation, gray/white matter volumes, fractional anisotropy, mean diffusivity, or whole brain cerebral blood flow among the 3 groups. As for DMN, the *ε4/ε3* group showed increased functional connectivities (FCs) in the left medial prefrontal cortex and bilateral posterior cingulate cortices/precuneus compared with the *ε3/ε3* group, and increased FCs in the left medial prefrontal cortex and right temporal lobe compared with the *ε2/ε3* group (*P* < 0.05, Alphasim corrected). No differences of DMN FCs were found between the *ε2/ε3* and *ε3/ε3* groups. FCs in the right temporal lobe positively correlated with the performances of vocabulary learning, delayed recall, and graph recall in all participants (*P* < 0.05).

*APOE ε4* carriers exhibited significantly increased DMN FCs when compared with *ε3* and *ε2* carriers. The *ε4* affects DMN FCs before brain structure and blood flow in cognitively intact young patients, suggesting DMN FC may serve as a potential biomarker for the detection of early manifestations of genetic effect.

## Abstract 2

Supplemental Digital Content is available in the text

## INTRODUCTION

Alzheimer disease (AD) is a progressive neurodegenerative disease. *Apolipoprotein E* (*APOE*) gene (*ε2*, *ε3*, and *ε4* alleles) is highly associated with AD.^1^*APOE ε4* allele is a well established genetic risk for decline of memory, *ε3* allele plays a neutral role,^1,2^ whereas *ε2* allele enhances neuroprotection against AD.^3,4^ Recent neuroimaging techniques, including functional magnetic resonance imaging (fMRI), allow for the investigation of genetic influence on the brain. fMRI studies showed decreased^5,6^ or increased^7^ default mode network (DMN) functional connectivities (FCs) and decreased hippocampal volumes^8^ in healthy middle-aged or older *ε4* carriers compared with noncarriers. Healthy older *ε2* carriers exhibited greater cortical thickness.^4^ All these findings support the fact that the effect of *ε4* and *ε2* allele on brain function and structure begins early in life.

Exploration of *APOE* polymorphism on brain function and structure in young cognitively intact individuals may help identify the early alterations before ageing leaves an imprint on middle-aged or older individuals,^9,10^ and better understand the pathophysiology of *APOE* on AD. Of limited fMRI studies in young *ε4* carriers, the structural and functional alterations varied widely: *ε4* carriers exhibited either no difference^11^ or alterations of hippocampal volume,^12^ and elevated^13,14^ or reduced activity^15^ during task-related fMRI compared with noncarriers. Majority of these studies recruited only *ε4* and *ε4* noncarriers^5,11,14^ and depicted no clear profiles of *APOE* polymorphism on brain cognition, structure, and function. Thus, we used multimodality MRI in cognitively intact *ε2*/*ε3*, *ε4*/*ε3*, and *ε4*/*ε3* carriers younger than 35 years to investigate the effect of *APOE* polymorphism on the resting-state brain function, structure, and blood flow.

## METHODS

### Participants, Standard Protocol Approvals, and Participant Consents

The local institutional review board of the Jinling Hospital, Nanjing University, approved this study. Written informed consent was obtained from all participants. A total of 215 cognitively intact adults were recruited from the local community between April and December 2013. Inclusion criteria were as follows: age 18 to 35 years; right-handedness; without any clinical diseases; and 12 years or more educational level. Exclusion criteria were as follows: any history of psychiatric or neurological illness; head trauma; drug or alcohol abuse; chronic corticosteroid therapy; diabetes; hypertension; magnetic resonance (MR) contraindications; and excessive head movement (more than 1.0 mm in translation or 1.0° in rotation).

### Neuropsychological Assessments

All participants completed a battery of neuropsychological tests,^5,11,12,14^ including measurements of cognitive states (Mini-Mental State Examination [MMSE] and Montreal Cognitive Assessment [MoCA] tests); attention/executive function (number connection test type A/B [NCT-A/B] and digit symbol test [DST]), intelligence (vocabulary learning and word fluency), Wechsler Adult Memory Scale (graph recall, visual recognition, touch test), and Wechsler Adult Intelligence Scale (similarities and digit span); and reaction capability (Chinese version of Stroop test) and depression (Self-rating Depression Scale [SDS]). The MoCA tests are associated with the domains of cognitive dysfunctions including executive function, naming, memory, language, attention, ability of abstraction, and orientation and delayed recall. Of MoCA tests, language, attention, ability of abstraction, and orientation were similar among the 3 groups; thus only executive functioning, naming, memory, and delayed recall were involved in further analysis. All the neuropsychological assessments were carried out by a trained doctor (XL) with 4 years of experience in neuropsychological tests, according to standard measurements as previously described.^5,11,12,14^

### Genotyping

The analysis of *APOE* genotypes was performed by an independent laboratory (Shanghai Tianhao Biological Technology Co. Ltd., Shanghai, China). Participants with homozygous genotype of *ε4*/*ε4* (0.9%, 2/215) were extremely rare and were excluded from the study. No participant with *ε2*/*ε2* genotype was found in the study population. Since *ε4* and *ε2* have been reported having different roles in AD processing,^16^ participants with genotype of *ε4*/*ε2* were excluded from this study. Of the remaining participants, 14 *ε2*/*ε3* carriers in *ε2* group, 31 *ε3*/*ε3* carriers in *ε3* group, and 31 *ε4*/*ε3* carriers in *ε4* group, matched for age, sex, and education level, were included for further analysis.

### Imaging Methods

#### Data Acquisition

Images were acquired on a 3-Tesla MR instrument (TIM Trio, Siemens Medical Solutions, Erlangen, Germany). A standard 12-channel phased-array head coil was used fitted with foam padding to reduce head motion. T2-weighted fluid-attenuated inversion-recovery sequence was used to detect clinically silent lesions (25 axial slices; slice thickness 4 mm; slice gap 1.2 mm; image matrix 232 × 256; field of view [FOV] 220 × 220 mm^2^; repetition time/echo time (TR/TE) 9000 ms/93 ms; flip angle 130°; inversion time 2500 ms; and bandwidth 287 Hz/pixel). Functional data were collected by using a gradient-echo echo-planar imaging sequence (all participants stay awake with their eyes closed: slice thickness 4 mm; slice gap 0.4 mm; image matrix 64 × 64; FOV 240 × 240 mm^2^; TR/TE 2000/30 ms; flip angle 90°, and bandwidth 2232 Hz/pixel). Each fMRI sequence contained 250 volumes, and each volume included 30 axial slices placed approximately along the anterior commissure-posterior commissure line. High-resolution T1-weighted images were obtained with 3-dimensional magnetization prepared rapid gradient echo (3D MPRAGE) sequence (176 sagittal slices; slice thickness 1.0 mm; no slice gap; image matrix 256 × 256; FOV 256 × 256 mm^2^; TR/TE 2300/2.98 ms; flip angle 9°; and bandwidth 240 Hz/pixel). Diffusion tensor imaging (DTI) was performed using single shot spin echo-based echo planar imaging sequence in contiguous axial planes. The original DTI images of each participant included 20 volumes with diffusion gradients applied along 20 noncollinear directions (*b* = 1000 s/mm^2^), and one volume without diffusion weighting (*b* = 0 s/mm^2^). Each volume consisted of 30 contiguous axial slices covering the whole brain (slice thickness 4.0 mm; image matrix 128 × 128; FOV 240 × 240 mm^2^; voxel size 1.8 × 1.8 × 4 mm^3^; TR/TE 4100/93 ms, and bandwidth 1396 Hz/pixel). Arterial spin-labeled (ASL) MR perfusion images were acquired using pulsed arterial spin-labeling sequence^17^ (slice thickness 5.0 mm; slice gap 1.0 mm; acquisition matrix 64 × 64; FOV 240 × 240 mm^2^; TR/TE 3000/11 ms; flip angle 90°; bandwidth 2232 Hz/pixel; delaying time 1200 ms; TI1 700 ms; TI2 1800 ms; and acquisition of 18 layers and a total of 90 time points).

#### Data Processing

Resting state fMRI data were preprocessed by using SPM8 (statistical parametric mapping, http://www.fil.ion.ucl.ac.uk/spm/) implemented in Matlab, Version 7.9 (MathWorks, Natick, MA). The first 10 volumes of the functional images were discarded to increase stability of the initial MRI signal and the adaptation of the participants to the environment. Images were corrected for acquisition delays (slice timing) before motion correction. The functional images of the remaining participants were realigned to the standard Montreal Neurological Institute (MNI) template (3 × 3 × 3 mm^3^) and smoothed by convolution with an isotropic Gaussian kernel of 8 mm Full Width at Half Maximum (FWHM) to decrease spatial noise.

#### Functional Activity and Connectivity Analysis

For amplitude of low-frequency fluctuation (ALFF) analysis, the smoothed data were detrended and temporally filtered to extract frequencies of 0.01 Hz to 0.08 Hz, then the ALFF map of each participant was extracted according to a previous fMRI report,^18^ using REST 1.8 software (http://resting-fmri.sourceforge.net). For DMN analysis, the group spatially independent component analysis (ICA) was performed to extract DMN on the smoothed fMRI data by using GIFT software (Vision2.0;http://icatb.sourceforge.net/). ICA separated linear mixed data into spatially independent components. To determine the number of independent components, dimension estimation of the smoothed data of the 3 groups was conducted by using the minimum description the length criterion, and the result was an average of 20 independent components. Then, fMRI data from all participants in each group were concatenated, and the temporal dimension of the aggregated data set was reduced by means of principal component analysis, followed by an independent component (with time courses and spatial maps) estimation by using the infomax algorithm. To each ICA, the time courses corresponding to the waveform of a specific pattern of coherent brain activity and the intensity of this pattern of brain activity across the voxels were expressed by the associated spatial map. Then, using the GIFT software (Vision 2.0d; http://icatb.sourceforge.net/), the DMN component of each participant was selected based on the largest spatial correlation with DMN templates provided by Dr Wei Liao (Center for Cognition and Brain Disorders and the Affiliated Hospital, Hangzhou Normal University, Hangzhou, China).^19^

#### Voxel-based Morphometry (VBM) Data Processing

Voxel-based morphometry (VBM) data processing was performed in VBM8 toolbox (http://dbm.neuro.uni-jena.de/vbm). First, high-resolution structural data were bias-corrected, tissue classified, and normalized to the MNI. Second, the standardized images were segmented as gray matter (GM), white matter (WM), and cerebrospinal fluid. Subsequently, analysis was performed on GM and WM segments separately, which was multiplied by the nonlinear components derived from the normalization matrix to preserve actual GM and WM values locally (modulated GM volume). Finally, all GM and WM images were spatially smoothed with an 8-mm FWHM Gaussian kernel.

#### Voxel-based DTI Data Processing

For DTI images, the 20 diffusion-weighted images were registered to b_0_ image using SPM8, and then corrected for difference in spatial distortion due to eddy currents using FMRIB Diffusion Toolbox (FDTv2.0) as implemented in FMRIB Software Library (FSLv4.1;www.fmrib.ox.ac.uk/fsl). Fractional anisotropy (FA) and mean diffusivity (MD) maps were then computed by using Diffusion Toolkit software based on our previous study.^20^ The b_0_ maps were standardized into a standard MNI space by using the echo-planar imaging template supplied with SPM8, which generated the deformation parameters that were applied to the normalization for FA and MD images. The normalized FA maps and MD maps were smoothed with an 8-mm FWHM Gaussian kernel.

#### ASL Data Processing

The cerebral blood flow (CBF) values in whole brain and regional brain were calculated within the ASL Toolkit (ASLtbx, http://www.cfn.upenn.edu) based on SPM8 software according to our previous study.^17^ Ninety ASL images were acquired from each participant, and the first one was taken as the equilibrium magnetization of brain (M0) image for CBF calculation. The first 2 pairs of ASL images were discarded during establishment of equilibrium of spin dynamics. After realigning, motion correction, and spatial normalization to MNI space, the 43 remaining of control/tag CBF image pairs were converted to mL/100 g/min using a single-compartment model. To eliminate the individual differences, the CBF images were normalized by using the equation CBF_corrected_ = CBF_uncorrected_/(GM + 0.4WM). Since the hippocampus is a key area associated with AD pathology, CBF values in bilateral hippocampus were extracted. Apart from the whole brain maps and 90 regional CBF maps, the bilateral hippocampus CBF maps, and the maps of the brain regions showing ALFF, DMN, FA, or MD differences among groups were extracted. The corrected CBF images were then spatially normalized.

### Statistical Analysis

The SPSS16.0 software (SPSS, Chicago, IL) was used for statistical analysis. Among demographic characteristics, neuropsychological scales, and the extracted MR imaging statistics, the qualitative data were described by relative ratio or percentage and tested by chi-square test. The normality of the remaining quantitative data, including age, education, and all the neurological assessments, were tested by Kolmogorov–Smirnov analysis. The data variance homogeneity of the 3 groups was analyzed by analysis of variance (ANOVA). In case of significant differences in the variance analysis, post-hoc analysis was used for intergroup comparison. In case of non-Gaussian distribution, the median and interquartile range [M (QU-QL)] represented, and k-independent samples nonparametric test was performed among the 3 groups. *P* value less than 0.05 was regarded as significant difference.

Amplitude of low-frequency fluctuation and DMN maps were analyzed for each group by 1-sample *t* test and corrected by false discovery rate (FDR) criterion with a threshold of *P* <0.05. ANOVA with Alphasim correction (http://afni.nih.gov/afni/docpdf/AlphaSim.pdf) was conducted among the 3 groups at the threshold of *P* <0.05, using SPM8 software. ANOVA was performed on normalized ALFF maps and DMN maps among the 3 groups by using SPM8 software in process Alphasim correction (http://afni.nih.gov/afni/docpdf/AlphaSim.pdf) at the threshold of *P* <0.05 with number of clusters of 79 and 121, respectively. If statistical difference was present, post hoc analysis was performed to detect the intergroup differences.

The ANOVA was performed on normalized GM maps and WM maps, FA and MD maps, and CBF maps (the whole brain, and regional CBF maps including bilateral hippocampus and the brain regions showing functional or structural differences) among the 3 groups, corrected by FDR criterion with a threshold of *P* <0.05. If the difference among the 3 groups were statistically significant, post hoc analysis was performed for intergroup differences. Bivariate analysis was conducted to analyze the correlation between brain regions showing functional or structural differences and neuropsychological scales. The data with normal distribution were analyzed by Pearson correlation analysis, whereas data with non-normal distribution was evaluated by Spearman correlation analysis with the threshold of *P* <0.05.

## RESULTS

### Demographics and Neuropsychological Tests

Table 1 shows the demographical and neuropsychological results. Age, sex, and educational level showed no difference among groups with *APOE ε4*, *ε3*, and *ε2* (*P* = 0.681, 0.988, 0.232, respectively). None of the neuropsychological scales showed any difference (all *P* > 0.05).

**TABLE 1:**
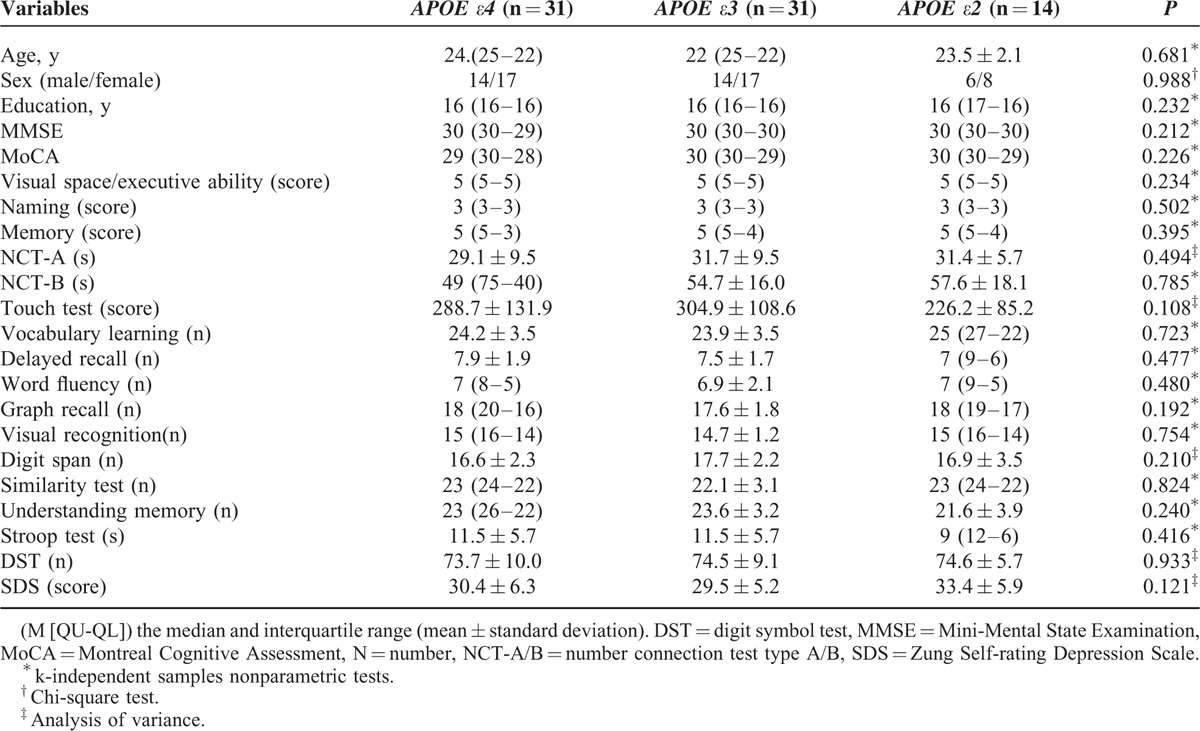
Demographic Characteristics and Neuropsychological Tests of 3 Groups

### DMN FC maps

**FIGURE 1:**
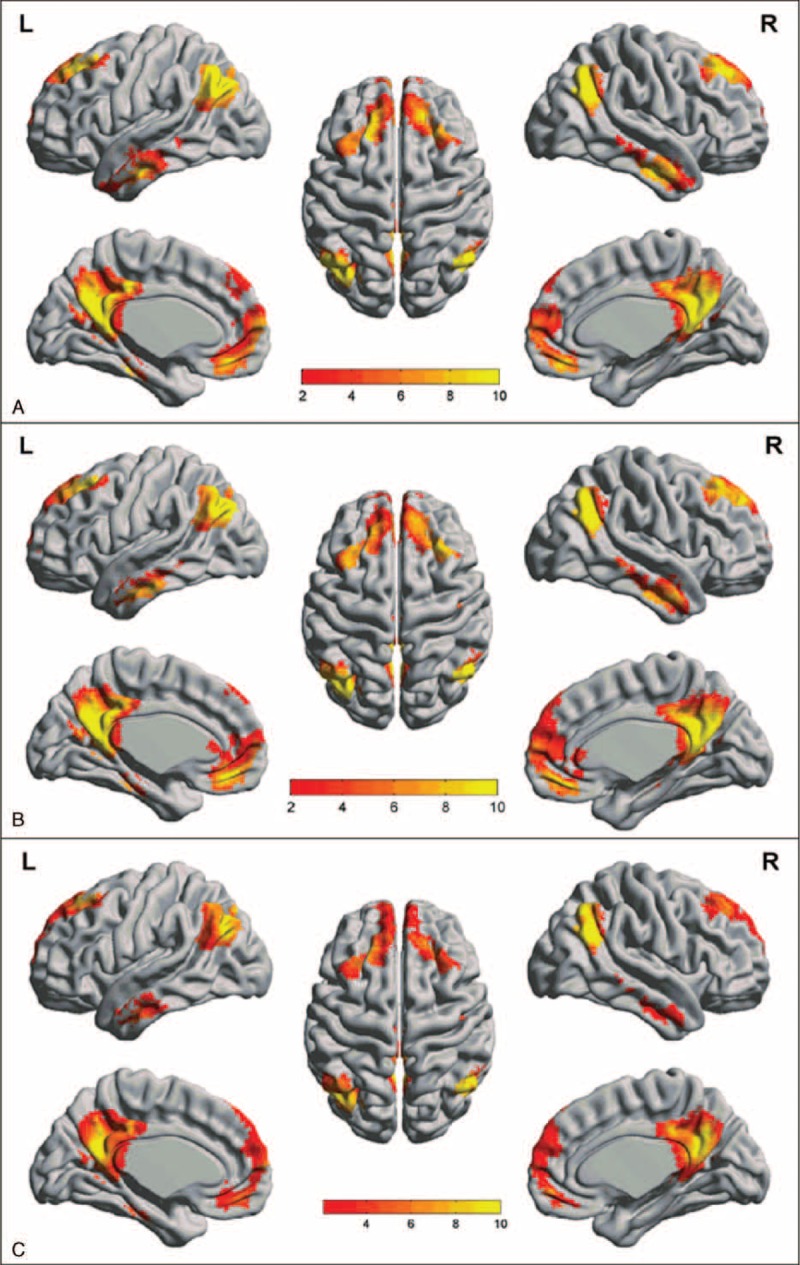
Spatial maps of DMN in *APOE ε4*, *ε3*, and *ε2* carriers. A similar DMN is shown from each group (A, *APOE ε4* carriers; B, *APOE ε3* carriers; C, *APOE ε2* carriers). DMN is mainly distributed in bilateral posterior cingulate cortices/precuneus, anterior cingulate cortices, medial prefrontal cortices, inferior parietal lobules, temporal lobes, and bilateral hippocampus and parahippocampus (FDR-corrected, *P* < 0.05). DMN = default mode network, FDR = false discovery rate.

Every group showed typical DMN spatial distribution pattern. DMN was mainly distributed in bilateral posterior cingulate cortices/precuneus (PCC/PCu), anterior cingulate cortex (ACC), medial prefrontal cortices (MPFC), inferior parietal lobules (IPL), temporal lobes and bilateral hippocampus, and parahippocampus (FDR-corrected, *P* < 0.05) (Figure 1).

**FIGURE 2:**
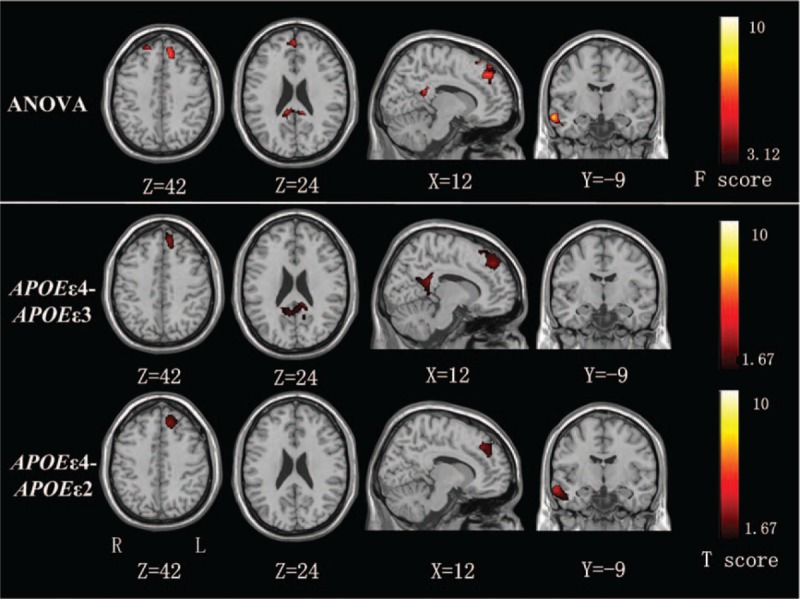
Spatial maps of the DMN by ANOVA among the 3 groups (*ε4*, *ε3*, and *ε2*). Spatial maps of the DMN shows that significant differences among the groups located in bilateral medial prefrontal cortices, posterior cingulate cortices, and right superior temporal gyrus. Post hoc analysis shows significantly increased functional connectivity in the left medial prefrontal cortices, and bilateral posterior cingulate cortices/precuneus in group *ε4* compared with group *ε3*, and increased functional connectivity in left medial prefrontal cortices and right superior and middle temporal gyrus compared to group *ε2* (Alphasim-corrected, *P* < 0.05). ANOVA = analysis of variance, DMN = default mode network.

Differences among the groups were mainly located in bilateral MPFC, PCC, and right superior and middle temporal gyrus (STG) and middle temporal gyrus (MTG). Post hoc analysis demonstrated that group *ε4* showed significantly increased FCs in the left MPFC and bilateral PCC/PCu compared with the group *ε3*, and increased FCs in the left MPFC and right STG and right MTG compared with group *ε2* (Figure 2 and Table 2). There was no difference between groups *ε2* and *ε3*.

**TABLE 2:**
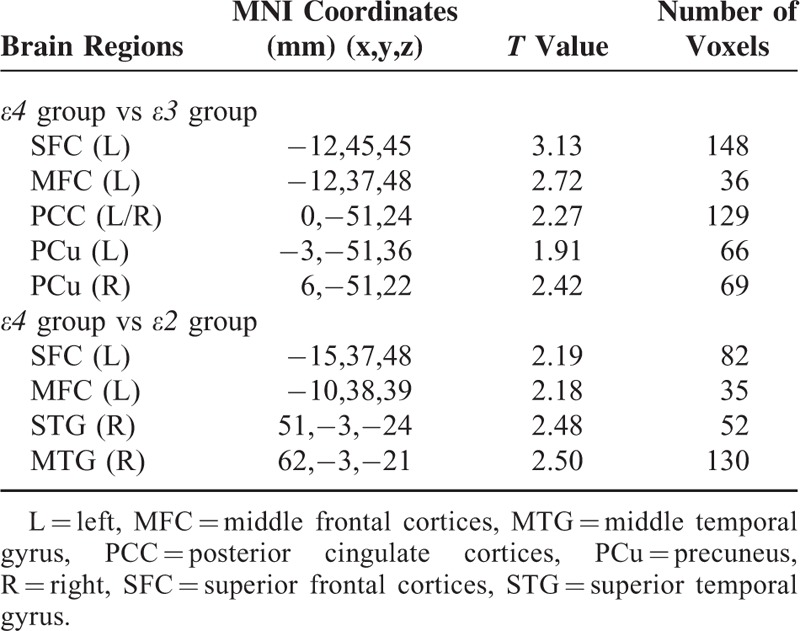
Differences of Functional Connectivity of DMN Among the 3 Groups

### ALFF maps

Results of 1-sample *t* test showed that ALFF maps were mainly distributed in the bilateral frontal lobes, temporal lobes, occipital cortices, cingulate cortices, PCu, inferior parietal lobules, thalamus, and midbrain among the groups. ALFF maps showed no significant differences among the 3 groups.

### Brain Volume Alteration

The volumes of GM or WM masks were no different among the 3 groups (Supplementary Table 1).

### FA and MD Maps

No differences in FA and MD values were found among the 3 groups.

### CBF Maps

Whole brain and each regional CBF showed no significant differences among the 3 groups, although the average CBF values in these brain regions showed a gradually increased trend of groups *ε4* < *ε3* < *ε2* (Table 3).

**TABLE 3:**
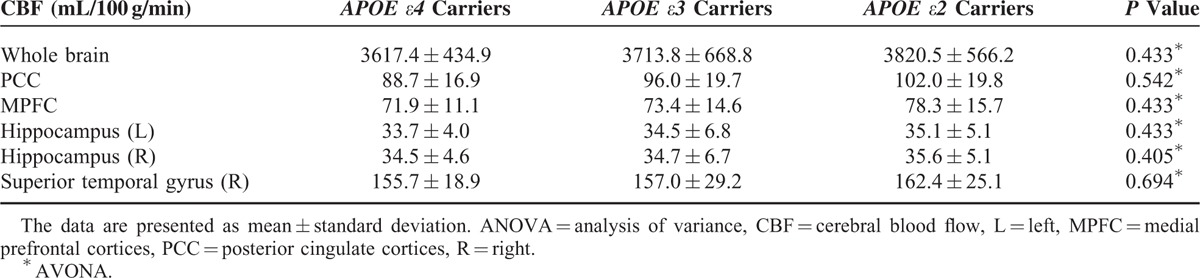
The Statistical Analysis Results of Cerebral Blood Flow Among the 3 Groups

### Correlation Analysis

Function connectivity in right STG positively correlated with vocabulary learning (*r* = 0.257, *P* = 0.025), delayed recall (*r* = 0.364, *P* = 0.001), and graph recall (*r* = 0.228, *P* = 0.048) (Table 4 and Supplementary Figure 1).

**TABLE 4:**
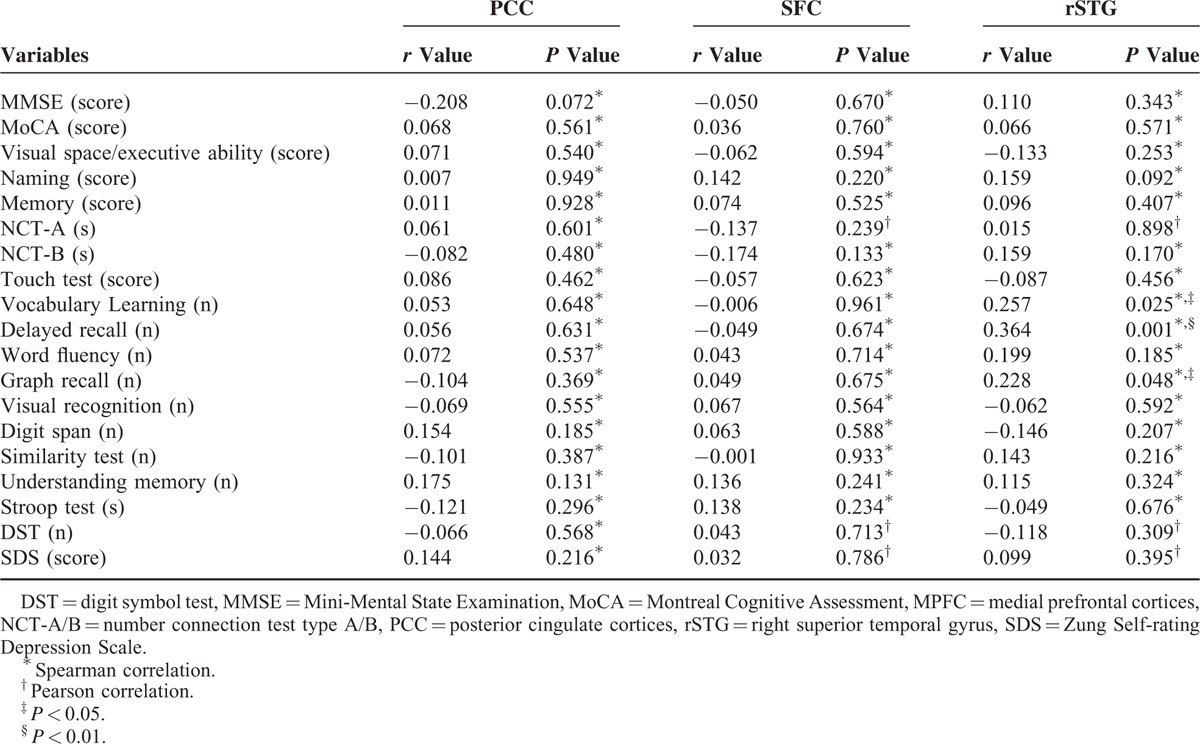
Correlation Results Between Brain Regions and Neuropsychological Scales

## DISCUSSION

Our study showed that *APOE ε4* carriers exhibited significantly increased DMN FCs when compared with *ε3* and *ε2* carriers. The *ε4* affects DMN FCs before brain structure and blood flow in cognitively intact young individuals. To our knowledge, this is the first study using multimodality MRI to investigate the influence of the *APOE* polymorphism (*ε4*, *ε3*, and *ε2* alleles) on brain structure, function, and blood flow in cognitively intact young adults.

In our study, *ε4* carriers exhibited significantly increased DMN FCs when compared with *ε3* and *ε2* carriers, which was consistent with prior studies^14^: *ε4* influences functional connectivity within the DMN before the onset of clinical disease in the youth. However, older cognitively intact *ε4* carriers manifested decreased DMN FCs.^21,22^ It is unclear whether these differentiations embodied in age-dependent alterations or potential pathological progress. The involved brain regions such as bilateral MPFC, PCC, and temporal gyrus were consistent with the preferential areas in AD.^22,23^ MPFC had been found to be linked with other components of the limbic system anatomically and functionally, and carries out self-referential mentations, executive function, and working memory.^24,25^ Both PCC/PCu and STG play leading roles in episodic memory.^26,27^ Positive correlation between the right STG FCs and vocabulary learning, delayed recall, and graph recall, which represent processes of productive and receptive memory, and consolidation^23,28^ was also reported. Our findings regarding the increased DMN FCs may be a compensatory for slight alteration of cognitive function in a very young age. *ε4* has a potential to disrupt the dynamic balance of excitatory and inhibitory neurotransmitters, which is crucial for learning, memory, and for DMN alteration.^29,30^ The increased FCs in DMN observed in this study might be the consequence of high glutamatergic concentration and/or low GABA compensation in the cognitively intact young *ε4* carriers. This assumption is supported by a recent study in which high regional inhibitory neurotransmitter levels were associated with enhanced deactivation, whereas high excitatory glutamate concentration was associated with reduced deactivation induced by the task.^31^ Thus, DMN might be an indirect biomarker for alterations of neurotransmitters in neurons and/or interneurons. In addition, the pathology of amyloid β disposition (*ε4* > *ε3* > *ε2*) and clearance (*ε2* > *ε3* > *ε4*), including synaptic deficits, mitochondrial dysfunction, and neuroinflammation in the brain^1,11^ in *ε4* carriers, might contribute to DMN alterations.

The DMN describes the spatial linkages between neuronal activities in different brain regions, whereas the ALFF measures the amplitude of the regional spontaneous neuronal activity.^13,32^ Both of these brain functional measurements showed abnormalities in MCI and AD.^33–35^ In this study, the DMN but not the ALFF showed abnormalities among the groups. The inconsistence between DMN and ALFF has been previously described in MCI and AD patients.^36^ One possible explanation was that alterations of spatial connectivity might precede alterations of neuron connectivity, that is, *APOE*-induced interneuron alteration precedes neuron alteration.

Voxel-based morphometry reflects brain macrostructure changes of gray matter and white matter. DTI reflects microstructure alterations of the integrity of white matter.^11^ Our study did not find any VBM and DTI differences among the 3 groups, consistent with a previously published study.^11^ Otherwise, 1 study found lower gray matter density in *ε4* carriers relative to *ε4* noncarriers via VBM in a wide range aged 19 to 80 years.^37^ However, it is unclear whether ageing or *APOE ε4* led to decreased gray matter density. ASL is able to noninvasively quantify CBF. In our study, ASL images showed no abnormalities in cognitively intact young *ε4* carriers inconsistent with previously reported results,^9^ in which young *ε4* carriers (23.6 ± 3.1 y) had increased CBF value in ACC. In this study, we observed the alteration of DMN before the alteration of brain macrostructure and microstructure, and CBF. The pathological mechanism of AD might help to explain the reason behind that functional alteration precedes structural alteration. White matter integrity has been reported to be positively associated with cerebrospinal fluid markers of amyloid-beta (amyloid β [42] and amyloid β [42]/p-Tau [181]) in AD.^38^ In a recent study, Tau protein has been reported to induce pathological changes in the brain including gray matter atrophy, increased white matter radial diffusivity, decreased amide proton transfer, and hyperperfusion in the rTg4510 mouse model of tauopathy.^39^ Whereas no differences in the concentration of plasma amyloid β peptides have been reported in young (28 ± 7.6 y) *ε4*, *ε3*, and *ε2* carriers without any memory deficits,^40^ suggesting that the alterations of brain macrostructure and microstructure, and CBF may not occur at an early age.

Even though *ε4* and *ε2* alleles may have different contributions to AD, *ε2* carriers manifested no significant alterations on brain function, structure, and CBF in this study. In recent studies, *ε2* carriers showed similar fMRI activity patterns with *ε4* carriers in patients aged 32 to 55 years^41^ and 60 to 80 years.^16^ The clear role that *ε2* plays needs to be further evaluated.

This study has some limitations. First, the frequency of *APOE* gene (*ε2*, *ε3*, and *ε4* alleles), especially that of *ε2* allele, is extremely rare. Additional studies with expanded sample size are needed to further investigate the genetic influence on alterations in brain macrostructure and microstructure, as well as CBF. Secondly, whereas the influence of *ε4*, *ε3*, and *ε2* alleles on brain function, structure, and blood flow was evaluated in this cross-sectional study, further follow-up studies are necessary to investigate whether *ε4* and *ε2* alleles have dynamic pathophysiologic changes along aging trajectories. Third, this research was focused only on sporadic ε4 carriers, and patients with AD-related family history need to be included in the future studies. Fourth, apart from DMN, other spatially distributed networks including executive-control network and salience network are needed to deepen the understanding of dynamic integration of early pathological manifestations and alterations of brain networks.

In conclusion, this study shows that the effect of *APOE* alleles on brain function precedes alterations in brain structure and blood flow in healthy young adults. DMN abnormalities may serve as potential biomarkers for detecting brain alterations in healthy young adults with *APOE* alleles.

## Supplementary Material

Supplemental Digital Content
