# Supplementary material for: APOE Polymorphism Affects Brain Default Mode Network in Healthy Young Adults: A STROBE Article
Source: Medicine (Baltimore). 2015 Dec 31;94(52):e1734. doi: 10.1097/MD.0000000000001734 (PMC5291594; doi:10.1097/MD.0000000000001734)
Supplement: Supplemental Digital Content [file medi-94-e1734-s001.docx]

**
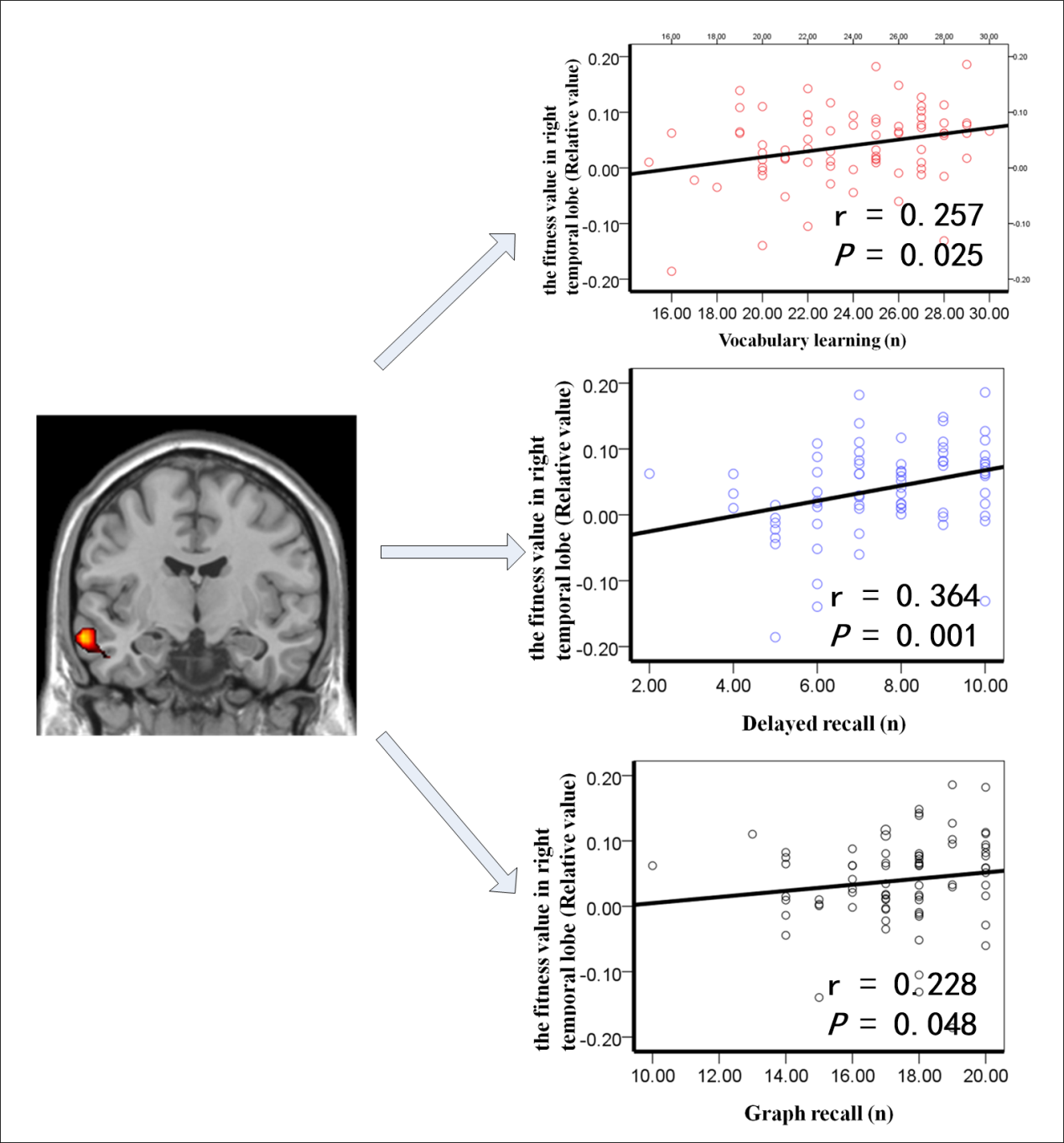
**

**Supplementary Fig. 1.Correlation results between the mean z value in right temporal lobe and** [**vocabulary**](http://cn.bing.com/dict/search?q=Vocabulary&FORM=BDVSP6) **learning, delayed recall, and graph recall (P < 0.05, Alphasim corrected).** [Vocabulary](http://cn.bing.com/dict/search?q=Vocabulary&FORM=BDVSP6)[learning](http://cn.bing.com/dict/search?q=learning&FORM=BDVSP6)[,](http://cn.bing.com/dict/search?q=%2C&FORM=BDVSP6) delayed recall and graph recall [were](http://cn.bing.com/dict/search?q=were&FORM=BDVSP6)[positively](http://cn.bing.com/dict/search?q=positively&FORM=BDVSP6)[correlated](http://cn.bing.com/dict/search?q=correlated&FORM=BDVSP6)[with](http://cn.bing.com/dict/search?q=with&FORM=BDVSP6) the [right](http://cn.bing.com/dict/search?q=right&FORM=BDVSP6)[temporal](http://cn.bing.com/dict/search?q=temporal&FORM=BDVSP6)[lobe](http://cn.bing.com/dict/search?q=lobe&FORM=BDVSP6)[fitting](http://cn.bing.com/dict/search?q=fitting&FORM=BDVSP6)[values](http://cn.bing.com/dict/search?q=values&FORM=BDVSP6).

**Supplementary Table1 - Quantitative brain volume comparison results among the three groups**

| Volume (ml) | *APOEε4* carriers | *APOEε3* carriers | *APOEε2* carriers | *P* value |
| --- | --- | --- | --- | --- |
| Gray Matter | 654.3±58.5 | 656.6±42.0 | 650.4±39.8 | 0.929^a^ |
| White Matter | 510.5±52.7 | 526.0±43.5 | 504.9±56.5 | 0.318 ^a^ |
| Cerebrospinal fluid | 225.1±27.6 | 218.3±23.4 | 215.5±24.9 | 0.415 ^a^ |
| Whole brain | 1389.8±128.0 | 1400.9±93.9 | 1370.8±122.0 | 0.714 ^a^ |

The data are presented as mean ± standard deviation

^a^ AVONA.
